# Educative Psychological Treatment at Edinburgh’s Royal Asylum: Unfolding *The Morningside Mirror*, 1845–1882

**DOI:** 10.1093/shm/hkae022

**Published:** 2024-05-01

**Authors:** Christopher Holligan

**Affiliations:** School of Education and Social Sciences, University of the West of Scotland, Ayr KA8 OSR, UK

**Keywords:** asylum, periodical, moral treatment, literature, science, class, insanity

## Abstract

This article examines moral therapy in relation to writing by fee-paying ‘lunatic’ asylum patients from the upper and middle classes. Their work was published in a nineteenth-century monthly periodical, *The Morningside Mirror*. There is an intersection of the periodical with status and the interests of gentlemanly values. Despite their psychopathological diagnoses, which included melancholia, writers for the *Mirror* retained their human capacity to share poignant insights into love and social injustice. Edinburgh’s reputation as a cultural and scientific centre of learning provided opportunities for the asylum to market itself as an iconic sanctuary that could maintain the materially privileged lifestyles of patients. *The Morningside Mirror* offered creative activity, self-esteem maintenance and public recognition. It connected the Asylum to the society outside. The expression of logic as reflective of the repair of reason signalled, from the viewpoint of psychological medicine, the *Mirror’s* therapeutic impact and utility to project reputation.

*The Morningside Mirror* was printed and published in Edinburgh’s Royal Lunatic Asylum as a monthly periodical. Its editors accepted patient contributions both from the Edinburgh asylum itself and from other asylums in Scotland and England. Its philosophy was associated with a Victorian culture that linked virtue with self-regulation. It was an ideal tool for delivering humane moral treatment interventions in a society that expected human emotions to be subject to the utmost self-restraint.^1^ Moral management was a mental science, a precursor to modern psychiatry in which the keeping of patient case notes allowed for the translation of moral treatment into practice.^2^ The *Mirror* appeared to offer asylum inmates a public intellectual space to have their voices heard amongst asylum peers and amongst members of the public beyond the asylum; it was also an opportunity for feelings to be conveyed to an imagined audience residing within their personal memories. Efficacy would no doubt also reside in opportunities for patients to engage with their delusional states and other mental symptomologies. A few readers may have wondered whether some of the many ‘biographical’ articles published in the *Mirror* might represent an accomplished narrative encryption of a patient’s case record rather than a lived empirical reality.

George Meredith arguably reflected this therapeutic opportunity in his 1871 novel *The Adventures of Harry Richmond* which narrates the troubled history of a family he was perhaps seeking to better understand. The novel examines what is known today as bipolar disorder, then classified as ‘circular insanity’, with the narrative focus following the title character’s self-destructive behaviour.^3^ Mania, dementia and melancholia were dominant diagnoses of most patients in Victorian asylums between 1870 and 1875 in England, where some case records identified all three in single individuals: ‘mania’ included grandiose ideas and ‘dementia’ involved cognitive impairment, with symptom overlap with modern schizophrenia.^4^ Mania was generally defined as being an agitated psychotic state, its description in nineteenth-century textbooks overlapping with twentieth century records where it includes euphoria, hyperactivity, grandiosity and flight of ideas including accelerated mental processes.^5^ Psychiatrists who treated mental disorders were known as alienists at this time because they cared for patients who were conceptualised as alienated from society and from themselves.^6^ Modern psychiatry recognises that a psychiatric disorder changes the patient’s view of themselves, their hopes for the future and their view of the world.^7^ Certain patients arguably compensate for feelings of alienation; some delusions might be indicative of the mind during periods of mental illness seeking different identities and roles that are inclusive.

Asylum psychiatrists at Morningside justified inviting patients to write for the *Mirror* on medical and moral grounds: the resident Physician-Superintendent Dr. Skae, who was in office from 1846 to 1872, announced its launch in grandiose terms: ‘a most philanthropic attempt at a new and rational system of mental recovery and improvement has been made … a powerful moral agent—for a great moral purpose—is even now slowly working its heaven-directed way in the cause of humanity’.^8^ An avenue to reach a cure in the Victorian asylum was the creation of a cultural and built environment that signified sanity; the *Mirror* contributed to that moral treatment construct. Diverse asylum environments were seen to impact maladies in diverse ways and the refined classes, possessing what were believed to be elevated sensibilities, needed to be catered for in a more refined manner.^9^

Inmates posted their contributions into an ‘Editor’s Box’ located within the asylum and designed for ‘voluntary offerings’. Subsequently these were reviewed by the editor to ensure they upheld certain standards: ‘Our Editor will take care that all which appears is connected with decency and good order at least, and as much like as possible’.^10^ Scattered footnote references throughout the *Mirror* name Dr. Skae as involved in accepting subscriptions, indicating his practical involvement in managing the periodical.^11^ A review of the *Mirror* by an imagined or real visitor’s thumb nail ‘advertisement’ asks readers to rethink prejudice and engage with its content:

Literature and the printing-press have, we believe, been of late introduced among the curative means of more than one asylum for insane patients, and they are now employed in the Royal Institution at Morningside, near Edinburgh. The Mirror, as we understand, is both written and printed by the patients in that asylum, and we should hope with the best effect. Amateur students of the science of the mind might be pleased to meet with the genuine, unpruned, and uncorrected effusions of persons labouring under various forms of mental disease; but there is nothing of the kind here … The preliminary … essay … would have made a respectable figure in Chamber’s Journal and the ‘Trip to Habbie’s Howe’ … might have graced those of our more ambitious Edinburgh prints which occasionally enliven and diversify their columns with sketches of local scenery … there is nothing startling, original, whimsical, or in the least eccentric about this new periodical; but the truth is, we can discover no other ground of complaint.^12^

Certain social fraternising taking place during sporting events at Morningside included the families of staff. The asylum used its own carts to transport large numbers of patients to Habbie’s Howe, a local beauty spot in the Pentland Hills. A patient signing as ‘A.P.’, who identified himself as ‘a happy inmate of the western department’, contributed an article to the *Mirror* describing one such outing.^13^ With fellow patients, he was invited by Dr Sherlock, ‘the younger of the three medical gentlemen of the establishment, to join a party of male patients’. Sixty inmates accompanied by ten attendants went by horse and cart: upon arrival they celebrated with the ‘basket of provisions’, ‘barrel of beer’ and ‘dinner on the grass in two rows’, as well as ‘cricket on the plain’ in a buoyant ‘great spirit’.^14^ The advancement of patients’ interests in natural history was one of the ‘distractions’ that accompanied moral management regimes in the work of alienists in Victorian Scotland.^15^ It was deemed a therapeutic recreation which was also recognised in accounts published in *The Morningside Mirror* of outdoor trips to nearby Blackford Pond.

Dr. Skae saw psychological treatment not as a mere mechanical application of psychological science but also as an artistic endeavour with an intuitive aspect. It was social and collaborative. In 1852, Dr. Skae expressed in the Asylum’s Annual Report his conviction that insanity arises from the breakdown of self-control.^16^ Skae brought to his employment a literary education gained at St. Andrew’s University before studying medicine at Edinburgh. He emphasised moral treatment in terms of patient labour, whether physical or mental. Moral treatment originated in the philosophical heritage of the Enlightenment’s faith in reason and self-control, emerging as a liberal psychiatric paradigm in Europe within a class mould whereby the wealthy were subject to different psychiatric theorising from the poor.^17^ The rich were believed at heightened risk because they relied more intensely on their minds whilst encountering greater anxiety by dint of their fastidious lifestyles. The predictable routines associated with physical toil protected the lower classes from ‘longings of ambition’.^18^ Madness was associated with sets of deficits.^19^  *The Morningside Mirror* attempted to decouple the notion of insanity from ideas of debilitation. For Dr. Skea, moral treatment should rest the diseased parts of the mind and, by engaging the patient in new trains of thought, revitalise the healthy parts of the brain.^20^ The therapeutic argument was that writing and publishing connected inmates not only to humane treatment and recovery, but also to the Victorian world of literature, politics, science and exploration.

Other than Emma Middleton’s conference paper and Vicky Long’s reference to *The Morningside Mirror* in her history of destigmatising mental illness 1870–1970, the *Mirror* has received little attention from historians.^21^ This article presents an exploration of patients’ voices in terms of subjects and themes. Whilst the register of a periodical differs from a personal letter it still provides an opportunity to engage with the intellectual and emotional interests of the asylum’s inmates. One exterior cosmos included significant amounts of fiction in periodicals reflecting on the social justice around class inequities in Victorian society.^22^ Edwards and Walker identify how the lifestyles of professionals in the nineteenth century signified class affiliation.^23^ Keeping alive memories through diary entries could maintain an educated person’s network of class and family intimacies, a practice identifiable, for example, in the life of a Victorian GP, Edward Wrench.^24^

Self-funding through periodical sales enabled ‘lunatic’ writers to explore and make public their cherished inner worlds. The *Mirror* fitted into an asylum space with an ambience of intellectual and social enquiry that promised the authors the chance of a role in conventions of publication that were available to people outside the asylum. The pattern of articles found in the *Mirror* paralleled the contents of periodicals in general circulation at this time. Supported by a growing collection of library resources, inmates of the Asylum are represented in the *Mirror* as rational interlocutors with the political, scientific, psychological (mesmerism, psychical research) and social concerns in the world outside.^25^ The symbolism of the presence of accessible erudition within its walls would undoubtedly augment and endorse the positive impression given about the therapeutic efficacy of moral treatment embodied in the *Mirror*. To establish a professional niche for psychiatry, alienists routinely promulgated assurances about their expertise.^26^

Victorian public libraries were, from their inception in 1850, presented by reformers and benefactors as productive of good citizens, ‘vehicles for social integration and harmonisation’.^27^ For some visitors to the Asylum, and readers who discovered the library’s existence through reading the *Mirror*, parallels with the spirit of the London Library might be conjured: acquired from 1841 the London Library’s book collection was instigated by Thomas Carlyle (1795–1881) whose book *Chartism*, published in 1839, had provoked debate about the state of the nation, and it was built up through donations, bequest and purchase. J. S. Mill (1806–73), whose own writings on politics, philosophy and economics connected radical thought with public life, recommended books to the London Library. His work of editing the quarterly periodical *The London and Westminster Review* also contributed to public debate.^28^ The Edinburgh Royal Asylum’s own library received donations from former patients, volumes of Thackeray’s ‘Ballads’ and a travelogue, ‘The Western Highlands and Islands of Scotland’, among them. George Stilwell, M.D. a member of the well-known Stilwell family, proprietors of the prestigious private asylum Moorcroft House at Hillington in Middlesex donated a modest £1. His obituary, published in the *British Medical Journal* on 10 August 1867, describes him as ‘the beloved physician to the establishment’.^29^ The Stilwell family carried on the tradition of cultured English aristocracy: Aldous Huxley was to become a close family friend.

Gillian Williamson argues that readers and contributors to periodicals fashioned themselves as ‘genteel’ and ‘inserted themselves as a public in the nation’s cultural and political life’.^30^ During the Victorian period ideas were engines of change.^31^ Interactions between science, politics and patronage had been established early in nineteenth-century Scotland, as Jenkins demonstrates in his account of the Royal Society of Edinburgh, 1800–40.^32^ Thinkers posed anthropological and religious questions, invigorating Victorian Britain’s intellectual life.^33^ Charles Dickens knew the editors of the *Edinburgh Review* in the 1840s which contributed to slavery debates in Parliament.^34^ Publishers of Sir Walter Scott expected readers of different social classes and tastes to be interested in this author’s work, so his writing was frequently published in differentiated editions to a variety of classes of reader.^35^ Besides politics, literature, medicine and travel writing about distant cultures were also pursued by those who contributed to *Blackwood’s Magazine*.^36^

The *Morningside Mirror* was an equally ambitious voice: it re-examined scientific reports on thermodynamics, the natural history of creation, T. H. Huxley’s model of science, theologies of health and disease, mathematics, the nature of humour and not least social prophecy.^37^ Against this background of widespread intellectual curiosity the asylum developed provision that normalised residency in this custodial institution, and contemporary physicians lauded the Edinburgh Asylum’s Reading Room as ‘a new source of pleasurable and useful recreation to the minds of those who incline to intellectual pastime’.^38^ Robert Lawson’s lectures to an Edinburgh Asylum audience of medical staff and patients, for example, explored human nature through humour, drawing upon Dickens, Sheridan and Samuel Johnston and classics in history and mythology.^39^ Upper-class Victorian physicians drew upon literary classics in their writing, incorporating characters as clinical cases; besides a clinical function, citations were a marker of cultural distinction that differentiated an esteemed scientific expertise.^40^ The conceptual associations of a medical culture with literature meant *The Morningside Mirror’s* project was situated within a supportive milieu.

This article aims to appreciate the context and the nature of moral treatment as reflected in the broad confines of *The Morningside Mirror*. The scope of the article’s emphasis falls upon the classist nature of patient contributions and how these can be conceptualised through the lens of moral treatment. The organisation of patients’ accommodation in the Edinburgh Asylum was overlaid with considerations of social class. Victorian Britain functioned through an intense consciousness of ‘place’ and status, and Asylum leaders could not ignore entrenched norms of class separation despite the common underlying reason for all the inmates’ presence in the Asylum. In articulating a project of mental healing, Dr. McKinnon, resident physician in the 1840s, recognised the social order in Britain in terms that the ideological architecture of the Asylum must respect prevailing social norms: ‘neither in the airing yards, nor grounds will inmates of the higher class mingle with those of the lower … their separation will be complete’.^41^ From the 1830s, the working-class was progressively being defined by processes of exclusion from privileges, not by virtue of a class volition.^42^ In 1880s Belgravia and Mayfair, wealthy Londoners left their elegant homes on dark evenings to crowd into omnibuses for midnight tours of the slums of east London, a voyeuristic process called slumming.^43^ There, elites experimented with fraternising with the working-class other, in locations that Charles Booth (1840–1916) the social reformer, documented street-by-street, in his colourfully coded sociological data storytelling cartography of poor and alleged criminogenic areas.^44^ Booth’s seventeen volume oeuvre *Life and Labour of the People in London* documented differences in the habits of various social classes in a discourse that linked poverty with depravity.^45^

Social class was also becoming biologised, as evidenced through class differentiated views about the brains of the mentally ill, including supposed class differences in the epidemiology of insanity evidenced in the ways asylum physicians implemented diagnostic schemes of symptom classification. Privacy and seclusion inside and outside the home were understood to be especially important to the middle classes: walls and boundaries secured their lifestyles.^46^ The asylum was, correspondingly, a socially layered house designed on a giant scale covering several acres in south Edinburgh. Space and detachment from neighbours were essential to class differentiation norms. In the Annual Report of 1888, the Asylum made the confident statement that it was a place ‘for patients of the richer classes’. The Executors of the late Mrs. Elizabeth Bevan, administering her huge financial bequest to the asylum made clear that it was for the relief of those insane persons ‘who, from their rank and society, or education and habits, cannot be associated with paupers’.^47^

## Social Class

Mid-nineteenth-century psychologists saw environment, experience and training as structuring neural pathways. T.S. Clouston, another of the resident-physicians at Morningside with years of experience in his role, added class to these factors, declaring that

the types of mental symptoms in the educated are far more differentiated and distinct. The lower you go in the social scale and in civilisation, the less distinct and complex are the types…To get a fine type of Melancholia, for instance, you must have an educated brain.^48^

Characters in the novels of Charles Dickens expressed concerns similar to those discussed by alienists regarding the stability of identity and the malleability of human nature to change.^49^ Elite public appointments protected a controversial scientific treatment zeitgeist: The Duke of Buccleuch and Queensferry was Governor of the Royal Edinburgh Asylum and his Deputy in that role in the early 1860s was Sir John Forbes. Sir John was a former navy surgeon in the colonies who recommended in his book (reviewed in the *Glasgow Medical Journal*, *The Athenaeum*, *The Spectator* and *The Examiner*) that medical practice should combine science with art to cure disease.^50^

What was the asylum’s demography that provided potential writers for *The Morningside Mirror*? The Royal asylums in Scotland accommodated private and pauper patients in a class-demarcated service where, besides accommodation and boarding provisions, diagnostic practices reflected class differentiation.^51^ The range of employments of the poor patients who lived in the Asylum’s pauper wing was varied: George Dickson, for example, admitted 6 May 1870 aged 60 years, was a joiner; Thomas Shuster, admitted 20 September 1878 aged 23 was a labourer; William Walls, admitted 4 April 1879 held a shopkeeper role; William Archibald, admitted 1 January 1880 aged 28, was a cook; Charles Young, admitted 8 March 1880 aged 36, worked as an upholsterer and William Beatie, admitted 7 April 1880 aged 45, had been a tailor.^52^ Common occupations as listed in the annual reports of those admitted were engraver, weaver, groom, slater, bricklayer, fisherman and bookbinder. Social class in the lunatic asylum manifested in multifarious ways: provisions and treatment reflected classist characterisations of susceptibility to illness which connected with state mandates to reform and manage the social order.^53^ The 1836 Annual Report on the Lunatic Asylum at Morningside recounts that the establishment of a Lunatic Asylum was proposed in 1792 by the Lord Provost and the Royal College of Physicians, but that events around the French Revolution slowed its progress. Its foundation stone was laid on 8 June 1809 carrying the inscription ‘An Asylum for the cure and relief of mental derangement’.^54^ Many patients admitted to the asylum in Morningside were diagnosed with ‘Melancholia’: in 1849 the total admitted was 265 of whom 26 suffered from Melancholia, 65 from Mania and 59 from Dementia.^55^ Forms of mania and melancholia were the most frequent disorders of those admitted and these inmates had higher recovery rates.^56^

The Asylum’s purpose, as presented to sponsors that year, was ‘That the Edinburgh Lunatic Asylum is intended for the reception of lunatics from both the higher and the lower classes of society’.^57^ Rates of board were determined by the wealth of the inmate with three different rates reflecting differing levels of accommodation and diet.

The professions of private patients included lawyer, medical doctor, bookbinder, teacher, stationer, professor, solicitor’s clerk, student in divinity, printer and bookseller. Others with private means were designated simply ‘Gentleman’ or ‘Lady’. One was a ‘Gentleman, formerly Brass Founder’ and another was an ‘Army Lieutenant of the Madras Army in India’.^58^ Not all originated in Edinburgh: Helen Hamilton Fulton, a 53-year-old Lady came from St Boswells, in the Scottish Borders; Helen Henderson, aged 27, from the island of Corfu; Jane Cooke, from Richmond, York; Dr Thomas Andrews, aged 40, from Belfast.^59^

In his 1841 Annual Report Dr. McKinnon argued that insanity was a ‘disease of the middle, or more active and useful members of life’ and that ‘material provision for the insane poor should differ from that appropriated exclusively to the wealthier class of patient’. He affirmed that ‘The scholar must have his library, the artist his studio, the agriculturalist his farm... An epitome of the world without, should be presented to them’.^60^ Status differentiation intersected with markers of class. Inmates beneath the ordinary rate were classified as ‘of an inferior rank’.^61^ And for superior accommodation, separate attendants and other extra comforts, a higher board was charged, which in 1849 ranged from £100 to £300 per annum, whereas in the Pauper Department (later renamed the Western Department) the fees in 1849 ranged from £19 to £24 per annum.^62^ The Eastern Department was described as the ‘higher class department’.^63^

When the creation of the Pauper Department had been originally proposed in 1829 objections were made about a plan to mix the classes of inmates; friends of the insane persons of the ‘wealthier classes’ opposed their being ‘housed with pauper lunatics’ and it was concluded they were to be housed separately, some within apartments with their personal servants.^64^ Other asylum providers interpreted amusements for patients in class terms, declaring those of the ‘superior orders’ as more suited to playing cards and the piano-forte.^65^ W. A. F Browne, Physician-Superintendent of the Crichton Royal Asylum, encouraged literary, musical and cultural activities.^66^ Different classes of patient in the Dundee Lunatic Asylum also received occupational and leisure opportunities that were geared to recognise their class and gender status.^67^ Class provision extended to the separation of inmates in the ‘refractory ward’ designed to hold violent inmates.^68^ Class differentiation included expenditure upon books, periodicals and stationery; in 1880 East House patients, numbering 123, benefitted from higher expenditure than inmates of West House, although the latter housed 716 patients.^69^

Jonathan Andrews identified that family refusals to agree to post-mortems in the case of deceased patients in the Edinburgh Asylum from 1870 were significantly higher among families of private patients, concluding social class was a factor affecting these objections.^70^ Questions of survival and personal immortality exercised the ruminations of educated Victorians; refusals to permit the dissection of deceased loved ones may have stemmed from a fear of disrupting their affectionate relations, or have reflected a view that it compromised an empirical afterlife.^71^

Victorian values shaped the construction of the causes of insanity.^72^ Inspecting Scottish asylums the 1857 Royal Lunacy Commission reaffirmed a metrics of class rank by recording the numbers of patients in the respective ‘Social Position’ of private or pauper, noting that the Edinburgh asylum held 176 private patients and 381 pauper class patients. This demographic profile meant it had plenty of potential writers for the *Mirror* to draw upon. Dr. McKinnon argued the ‘exciting causes of the disease—moral and physical—are in the most powerful in society’ during the ages of 20–50 years.^73^ Further he declared that ‘an Asylum ought not to be a prison, and we contribute to divest it of such a character, by allowing its inmates to see the face of nature beyond its walls’.^74^Most inhabitants in the Asylum were certified patients, rather than voluntary inmates, which helps to explain sentiments about the need for supervision.

## *The Morningside Mirror*: Rationales and Content

Dr David Skae, resident Physician-Superintendent, advocated asylum experience as ‘a massive engine of distraction’ to return patients to a normality from which they had been severed by pathology.^75^ The Asylum Admission Register for the period 1817–63 might give rise to questions about the efficacy of ‘distraction’ in the light of serious diagnoses of ‘monomania of pride’, ‘epileptic mania’, ‘general paralysis’, ‘melancholia’, ‘moral insanity’, ‘monomania of suspicion’, ‘acute mania’, ‘suicidal melancholia’, ‘monomania of superstition’ and ‘monomania of fear’.^76^ Nevertheless the launch ‘Address’ in the first issue of *The Morningside* Mirror, signed by patients J.F.R. and G.P., expressed the view that mental illness can in some cases co-exist with literary capacity.

…to a certain class our remarks cannot be held to apply. We allude to those melancholy cases that are utterly hopeless, and in regard to which, therefore, it becomes us to remain silent…they have been placed beyond the pale of human interference. In regard to those, however whose intellect may be only somewhat defective, or slightly impaired, we have at present daily opportunities of observing the wholesome stimulus which is produced by active bodily employment.^77^

This philosophy was embodied in the periodical which sold for three pence in Edinburgh booksellers in Princes Street and Howe Street or through mail delivery or subscription orders handled by resident physician Dr. Skae.^78^ Profits from the sale of the Morningside Mirror were invested in improving the Asylum Library. There appears to be some likelihood that the clerk to Sir Walter Scott when *Waverley* was published may have supported the Mirror’s production.^79^ The 1853 Annual Report recorded the cost of printing the Mirror monthly from January to December was £18.7.0; reprinting back numbers cost £4.10.0.^80^ In the 1851 address ‘*To the Courteous Reader*’ the editor of the *Mirror* challenged entrenched attitudes to the care of ‘lunatics’, recognising that ‘madness’ was thought to undermine agency. It was an unusual entry to a competitive publishing market: ‘The idea of a Periodical emanating from a Lunatic Asylum, and exclusively written by madmen, was certainly a bold and novel conception’.^81^ This edition of the *Mirror* set out its goal for ‘intelligent patients’ and an invitation to ‘benevolent minds’ to empathise and support them:

The great and beneficent purpose has been attained, that of affording to intelligent patients a field of useful literary exertion, which may not only contribute to their own, but may be instrumental in diffusing a feeling of sympathy in benevolent minds with their unfortunate position.^82^

The *Mirror’s* editorial reaches out, urging a caring population to come alongside this class of patient. The contents of the *Mirror* raise questions about the assumption that madness diminishes the humanity of individuals, a theme also explored and interrogated in Victorian literary fiction.^83^ Writing for a periodical, unlike writing for newspapers in the early nineteenth century, was considered a suitable occupation for gentlemen. The editorship of the *Quarterly Review* was a post sought by acclaimed literary authors as it gave them access to its readership among the elevated social circles of the educated classes; journalists, by contrast, tended to voice the views of the people, so, in a country run by aristocratic networks, were treated with some suspicion or even hostility. William Jackson, an editor of the *Morning Post* in 1795, who was discovered to be a French spy, had not been forgotten.^84^

Subjects addressed by authors whose writing was published in the *Mirror* included classical civilisation and historical analysis, travel and exploration, wars and military conflicts, social justice, literature and poetry, religion, science, psychology, grief and memoirs; asylum news and accounts of outdoor trips, lectures and entertainment, and reports by asylum physicians also featured regularly. The asylum’s librarian, who was a patient, undertook various literary and clerking operations in the library which was seen as a ‘constant source of intellectual improvement’.^85^ Dr. Skae described the vibrancy of the *Mirror* three years after its launch:

The *Mirror* continues to be written for, and to be read both in the House and abroad. The profits of its sale afford a handsome supply of newspapers and its circulation keeps up an interest in the Institution which has led to the reception of many handsome donations of books.^86^

The production values of the *Mirror* and the authenticity of its outputs was emphasised under the rubric ‘Amusements’; presumably most of this work was undertaken by the fee-paying East House patients who were described as ‘the intellectual class’^87^ and ‘the middle class of society…Many of them are persons of culture, who have been accustomed to a life of comfort and refinement’.^88^ Dr. Skae celebrated the Mirror’s success in establishing a place in Asylum life:

The monthly periodical ‘The Mirror’ is now in its sixth year and continues to afford occupation and amusement to an increasing number of contributors and readers. It chronicles many of the more interesting events of the House and the contributions of friends to the Library. The profits drawn from the sale of it are devoted to the purchase of periodicals, music, and occasionally new works of interest.^89^

…the Monthly periodical, written by the patients, has been entirely printed by them, with the exception of one or two numbers. The circulation of our little journal has been extended, and all profits continue to afford a liberal supply of newspapers and periodicals.^90^

Seven years later educated professionals, upon their inspection of the asylum for the Royal Lunacy Commission 1857, were impressed: the ‘treatment adopted towards the educated classes is … very praiseworthy’. They saw reason to include recognition of *The Morningside Mirror* and the asylum’s independent press: ‘… at Morningside, periodical publications are regularly printed and circulated, many articles being contributed by the patients themselves’.^91^

The *Mirror* clearly held a recognised status in the asylum’s moral treatment regime. It was an ‘Amusement’, but also a channel for the voices of those capable of writing. Periodicals had shifted their target readership beyond well-connected gentlemen in a nationally competitive market.^92^ In 1846 a *Mirror* editorial announced that ‘The object which the Directors had in view, when they put the Press in motion within these walls, was to afford the sedentary and the studious an opportunity of employing a portion of that time so much at command in pursuits congenial with former habits’.^93^ That congeniality had to be protected: patient contributions published in the *Mirror* did not seem to reflect the delusional worlds described in the Asylum’s case books and in some patients’ letters to the outside world. None of the psychopathology found in patients’ letters by contemporary psychiatrists was apparent in pieces in the *Mirror* for a less intimate audience. The periodical offered limited recognition of individual suffering or knowledge about symptoms and behavioural manifestations of patients’ recalcitrant clinical pathologies.

Writing capacity is related to social class and level of educational background and the possession of a dip pen communicated social class distinction.^94^ The 1841 Annual Report notes the ‘well educated’ as numbering 13 inmates, and patients with the ability to read and write numbered 21 inmates.^95^ The editor of Volume 1 (1845–46) of *The Morningside Mirror* claimed that ‘This little periodical, printed in the Asylum, is entirely the work of inmates’ and has ‘moved some to exercises who before were listless and indolent’.^96^ It was sold in Edinburgh and distributed to other asylums, but its readership and revenue were likely to have been modest. In 1845, the first issue of *The Morningside Mirror* was reviewed in Tait’s *Edinburgh Magazine*.^97^ The *Edinburgh Review* and *Blackwood’s Edinburgh Magazine* amplified Edinburgh as a ‘voice in the land’.^98^  *Blackwood’s Edinburgh Magazine* promoted the importance of feeling, empathy and the imagination, themes that often recurred in the *Mirror*.

Besides the drop-box delivery of writing, the practice of using a pseudonym meant true-life identity of *Mirror* authors remained a mystery.^99^ From around the mid-eighteenth century newspapers used ‘subtle methods to encourage their readers to believe that they spoke with the voice of the people’: pseudonyms were employed to indicate that writers were not speaking for themselves, but as representatives of the views of a social group.^100^ Contributions to the *Mirror* privileged self-control, work labour, outdoor agency and the serious traits admired by elites.^101^ Unlike the content of *The Morningside Mirror,* Letters and Annual Reports, as well as admission registers and patient case books, contained data about psychopathologies which might well have confirmed the social prejudice that an asylum was a place of terror, had they been publicly available.^102^ Vicky Long suggests contributors to the *Mirror* used linguistic strategies that playfully inverted hierarchies of social and political power. She claims the *Mirror* gave ‘some asylum patients a forum in which to forge a different identity for themselves than that of mental patient, a place to discuss literary genius, exotic locations and historical events; to escape the humdrums of asylum life’.^103^ Some, rather than forging different identities, used their writing to re-examine their feelings and attachments.

The contributions of ‘H.M.,’ included seventeen pieces between 1851 and 1857, rising to forty-one between 1857 and 1863, but of course H.M. might have been initials used as a pseudonym by many writers. Other pseudonyms included ‘Omega’ and ‘Der Englander’ (1845–46) ‘Eureka’ and ‘Chirurgicus’ (1851–57), ‘Philosoph’ and ‘Fankwie’ (1857–63). An author signing him or herself ‘J.C.’ seemingly published 19 pieces between 1845 and 1863. Another pseudonym, ‘Iran’, was associated with 80 published pieces between 1851 and 1857 followed by a further 23 between 1857 and 1863. The patient or patients signing as ‘L.D.’ had 6 pieces published between 1845 and 1846, 43 between 1851 and 1857, then a further 5 pieces from that point until 1863.

## Melancholia and Grief

Melancholia together with mania and acute mania are the two most prominent disease categories found in case books, admission records and annual asylum report statistics. The majority of ‘suicidal lunatics’ in British asylums were diagnosed as suffering from melancholia, judged a risk factor for suicide and morbid tendencies.^104^ Of 248 patients admitted to the Edinburgh asylum in 1853, 30 were recorded as suffering from ‘Melancholia’, some of whom were also among the 57 admittees with a ‘Suicidal Tendency’.^105^ In the annual report of 1880, 185 inmates were recorded as suffering with some form of mania and 114 with melancholy, out of a total number of 347 patients admitted in that year. Moral treatment was designed to replace a ‘listless and inactive mode of life’ and heal the patient by distracting ‘the current of his thoughts’.^106^ Moral treatment seemed, as framed, to have partially worked: in 1880 melancholic patients had comparatively high rates of recovery, but on the other hand, suicidality was exceptionally marked among those with the disorder, as evidenced by annual report data.^107^ Dr. McKinnon categorised melancholia as ‘gloomy forebodings … the … wish to terminate their miserable existence by suicide’.^108^ The ‘passions’ were seen to play a role in insanity; the persistence of grief, for example—which can extend to loss of attachments to an earlier life, a business, or home, as well as to people—meant individuals kept intimate attachments alive despite the object of love being lost to contact.^109^

Asylum patients writing about the emotion of grief and loss in *The Morningside Mirror* were retaining and re-establishing emotionally important ties with others. Expressions of grief in nineteenth-century literature are embedded in a Romantic ethos where the pain of grief appears tolerable, and embraced as personal and intimate.^110^ Expressed in the first-person pronoun, grief is revealing of the writer’s mental state and social relations. Its introspective basis indicates the writer has reflected on their emotional state and can now recognise and communicate that grief is the emotion identified.^111^ For nineteenth-century physicians, in ‘melancholia the tone of the mind was slackened and subdued. The mental depression was one in which the operations of the brain were dampened, lowered’.^112^ The resident physician in 1849 recorded the instances of ‘suicidal tendency in those admitted’. Of the 61 patients admitted that year, the majority were diagnosed with melancholia.^113^ Presentations of melancholia, represented in loss, isolation, longing and sadness in the voices of inmates are illustrated in the following two poems, both published anonymously in the *Mirror*.

*    SLEEPING*
When the fancy roams in the lone midnight,
The friends whom I loved appear to my sight;
The illusion departs with the dawn of day;
Like the mists on the mountain they vanish away.
In my dreams, I kneel down in the House of the Lord,
With hope in my bosum, and faith in His word;
But the night wears away, and my dreams are dispell’d,
I awake but to feel I’m a prisoner held.
In my dreams I recal youth’s happier day, -
I awaken – the vision has melted away-
While the gloomy hours, in their course so slow,
I reproach – but my grief it is in vein to shew,
Were I free to change, I would gladly roam.
From the haunts of men to some cottage home,
Where the sounding stream, and unwavering breeze,
And the feather’d choir ‘midst the shady trees,
And the sun of truth, with its purist beams,
Would enliven thought and direct my dreams.^114^

*    To I ----- F------*
Congenial soul, when next we meet
We’ll taste of love the waters sweet;
Wandering thro’ the bowers of bliss,
And prove the fountains pure and deep,
Where truths unknown a silence sleep…
We’ll learn the music of the spheres,
And trace his works through endless years.
In silent darkness let me lie,
At least, all that of me can die.
Alike for getting and forgot,
And save by the remember’d not.
Love strong as death doth clear the gloom,
To part with thee was worst of all;
The love I feel will last for ever.^115^

Poetry of this character, engaging with grief, loss and intelligible pessimism, is scattered throughout the *Morningside Mirror*.^116^ In the first poem a suffering inmate experiences the asylum as an incarcerating gloom which is punctuated by relief given through escaping into thoughts of something cherished, but now inaccessible. This poet’s mood conjures longing and a sense of exclusion from the society with which they are still connected emotionally. The second poem describes the pain of incarceration and the gradual disappearance of the author’s human wellbeing as they subsist in a ‘silent darkness’ while time passes but an emotional connection remains strong. The mood and content of each poem reflects the sentimental literature then popular outside the asylum. The selfhood of each patient reaches and connects with us as readers despite the presence of a material and symbolic stultification of their custodial setting infused with psychopathological meanings. Each poem conveys anxieties about human vanishing amidst a sense of personal decay. Other patient voices in the *Mirror* articulate and defend political values, acting as spokespersons on behalf of social classes or examining principles of justice to highlight oppression.

## Social Injustice

The 1840s and subsequent decades in Britain were characterised by political protest fuelled by urban poverty both of which stimulated the emergence of Chartism in a context we learn more about through ‘condition of England’ novels by Dickens and his contemporaries.^117^ In London’s workhouses paupers challenged the disciplinary apparatus of the new poor law and were committed to prison for insubordination and protest.^118^ During the nineteenth century, Lockean radicals addressed problems of liberty and equality generated by the growth of industry; on the one hand ‘libertarian’ thinkers argued that the world of resources should be available to whoever can take it, because it was originally unowned; they regarded the state’s redistribution of wealth as without justification, except to correct past injustices.^119^ The ‘equalitarian radicals’ by contrast argued the state should employ redistributive taxation, accepting a common right to share equally the world’s resources. Class conflict between labour and capital characterised the hostile manipulative behaviour of, for example, the anti-trade union owners of the company Staveley Coal and Iron Company Ltd during 1863–1900 who, besides deceitful reputation management, denied workers access to accounting information relevant to their wage bargaining demands.^120^

Outside political registers of social analysis, themes of social injustice in literature pervade Charles Dickens’ novels which examine class hierarchies situated within coercive and harmful structural inequality.^121^ In *Bleak House* Lady Dedlock is depicted as a victim of social injustice concealed by Victorian moralising hypocrisy. In *Great Expectations* Pip is also the victim of brutal social injustice. Novels acting as vehicles for commentary and social critique also included Dickens’ *Hard Times* (1854), Benjamin Disraeli’s *Sybil or the*  *Two Nations (1845)* ^122^ and Elizabeth Gaskell’s *North and South* (1855). Gaskell’s contribution is a ‘condition of England’ novel set during the period’s social turbulence when social inequalities of class and wealth shaped imbalances of power, colouring the tensions present in industrial conflict as capitalism developed.^123^ The legal establishment in Dickens’ oeuvre is corrupted by a class prejudice perpetuated by brutish self-seeking and strategic deceit. In the Chancery world of *Bleak House* (1852) everyone lives in webs of acquisition and human exploitation in what Dickens saw as a diseased, faceless bureaucratic system.^124^

Chartist newspapers’ critiques extended to Britian’s colonial state, calling for the abolition of slavery and highlighting the disenfranchisement and impoverishment of the working classes.^125^ At the height of empire, discourses about superior moral character were used to justify Britain’s colonial grip on its foreign possessions.^126^ Following mass starvation in Ireland during the 1840s, hunger was still pervasive among the people of England in the mid-nineteenth-century: despite Malthusian prophecies hunger was a constant and feared presence in working-class life.^127^ R.W.s critical, political, indignant voice, published in *The Morningside Mirror* in 1847, pours scorn on Malthusian politics as antagonistic to the wellbeing of the working classes:

*             England in the Olden Time*

The political economists of the Malthusian school are particularly fond of drawing parallels between the comforts of the working classes *now* and in the ‘Olden time’. Some of them do not hesitate to give *false* statements to maintain and prove their favourite position. We deny that the comforts and enjoyments of the working classes, viz. the great mass of artisans, weavers, agricultural labourers, etc, have increased in proportion they are entitled to expect from the advance of scientific improvements, and increase in machinery and capital. That their general condition has deteriorated since 1815, while the amount of manufactured articles have more than doubled…When competitive machinery has reduced useful craftsmen to poverty, a class parliament tells them they ‘must fall back upon their own resources’. In the name of all that is just and good, we would ask what ‘resources’ has a family man … to fall back upon?^128^

Themes of class division and structural injustice extend into an interpretation of crime in an essay by the patient signing as ‘W’:

*               CRIME*

We believe it is Addison who compares a human being without education to a block of marble in the quarry – a shapeless mass till moulded into form by the hand of the sculptor. More money has been expended in this country in the trial and punishment of crime, than would educate three times her population – for what end we ask? For the maintenance of a class to whom the highest offices of the state are open, and whose status in society chiefly depends on the vices and follies of others … Where misery abounds, there delinquency is fostered; the parent teaches his child to despise those laws which have not kept famine from his hearth … the rapid increase in crime is beyond their control; it is a national evil, and hence the necessity of natural interference, but a state education is not antidote against social corruption, if not enforced by moral and religious precepts.^129^

The values of moral treatment were translated by editorial judgement into enlightened commentaries regarding the harms and adversity which diagnostic theorising by the asylum’s physicians recognised as an environmental source of mental instability. The next section engages with a different milieu, where the meanings of articles published in the *Mirror* point towards a documentation of settings and social resonances which nurture and deliver a form of moral treatment experienced by osmosis. The contents of these writings would also, as indicators of esteemed cultural capital, hold the attention of wealthy families who formed an important income stream for the Asylum. Their fees enabled West House’s financial viability and drove estate development at Craig House and at a villa near Cockenzie which was a seaside retreat for ‘successive parties of ladies and gentlemen’ in the summer months.^130^

## Character and Moral Integrity

Travel and meanderings that extensively indulged in descriptions of exotic landscapes and adventure overseas pervade the pages of *The Morningside Mirror*. As renowned upper-middle-class literary artists sought exotic retreats for their mental recovery, so travel and wealth and networks were required. Journeying slowly for days pulled along by horse and carriage was part of a pilgrimage ushering in different selfhoods. Novelists R.L. Stevenson (1850–94) and Arthur Conan Doyle (1859–1930) set up homes in the Swiss village of Davos. Leslie Stephen (1832–1904) literary critic and father of Virginia Woolf scaled formidable Alpine peaks. English mountaineer Edward Whymper, after several attempts at scaling the 14.690-foot Matterhorn, reached its summit with six companions including an English aristocrat on 14 July 1865. On descent one member of this adventurous group slipped and fell pulling the others attached to the same rope down with him; the rope broke under the strain and four members of the party, including Lord Francis Douglas, plunged to their deaths. The tragedy generated extensive coverage in the British press.^131^ The European Alps were romantically perceived as liberating gateways to one’s psychological essence.^132^ Writing about character in nineteenth-century American literature, Susan Ryan argues that human character was ‘something to be achieved and maintained through the energetic cultivation of good habits and the relentless rooting out of bad ones. Just as crucially, character had to be communicated to the world beyond the individual self’.^133^

The communication of virtuous character is illustrated by patient Peter Daly Murray whose narrative contribution, as P.D.M., to *The Morningside Mirror*, published on 16 October 1848 and entitled *A Sea Fight*, is possibly an encryption of one of several delusional ideations.^134^ Peter Daly Murray is one of the few patients the establishment of whose identity as a contributing author to *The Morningside Mirror* is reasonably secure. There are several pages of case notes about Peter Daly Murray who experienced a ‘classical education’, was possessed of ‘superior abilities’ and had a diagnosis of ‘acute mania’.^135^ He had been a ship’s surgeon with H.M. Navy for 20 years, serving in Africa and the West Indies. Besides believing himself to be the heir of the title and estate of the Dukedom of Athol, the current incumbent of which he judged to be an imposter, he believed that packages aboard a steamer in which he was travelling to Campbeltown contained arms intended to ‘assist French Revolutionary Republicanism or disaffected Irishmen in rebellion’.^136^ In the exercise space of the asylum airing grounds he called on troops to surround the house, later believing that marks of nails on his hands proved he was the Christ, and he suffered paranoid thoughts of persecution: his case notes record he was on some occasions held in seclusion and ‘displays much suspicion. Fancies that his visitors are foes, that his food is poisoned’.^137^ P.D.M.’s tale in the Mirror begins with a didactic poetic framing of virtue: ‘All desperate hazards courage do create, He who acts boldly is of great estate, Presence of mind and courage in distress, Are more than armies to secure success.’ The incident on the ship described in his article published in the *Mirror* is an attempted mutiny by French imprisoners of war on the ship where he held a post of command as captain or the Lieutenant A.M who observed suspicious behaviour on the voyage:

During the voyage suspicious circumstances occurred to verify the belief already entertained by Lieutenant A.M. that the prisoners would rise to take the ship. A Frenchman, a kind of valet and hairdresser employed by the navel surgeon and his assistant was observed to be continually peering into the small cabin where the arms were kept…The evil, though checked openly, was continued secretly, and the Lieutenant M’s vigilance redoubled ….

The narrative that continues is emblematic of an elite Victorian typology of manly virtue, the ship is saved and the hero is publicly recognised by the Duke of York, the commander-in-chief, for his gallant conduct.^138^ Besides the conjuring of messages of meritorious worth and virtue embedded in martial conflict, other subjects in the *Mirror* hold threads of moral redoubt playing into the doctrine of moral treatment medicine which its readers can assimilate.

The scientific revolution of the seventeenth century instituted a moral awakening around cultures of enquiry. The spirit of the new inductive science was upheld through virtues of self-discipline, courage, patience and humility.^139^ The aesthetic force of Victorian critical prose was conditional on its ethical values, exemplified in the use of virtue terms derived from Aristotelian ethics by eminent critics such as John Ruskin and Matthew Arnold.^140^ A patient writing about the Covenanters in the November 1845 edition of *The Morningside Mirror* highlighted the notion that armed conflict was resolvable through courage. Individuals making the sacrifice became cherished martyrs who had given their lives for the greater good:

The memories of those who, with true heroism of martyrs fought and fell in such a cause, can never cease to be regarded by Scotchmen with feelings of the deepest reverence … several … fought with their broad swords in one hand, and Bible in the other. A more sublime instance of sincere devotion to the cause which they maintained can scarcely be imagined.^141^

Covenanting memory is founded on piety and principle, resisting persecution with force of arms: many nineteenth-century texts articulated Scotland’s debt to the Covenanters.^142^ Throughout the Victorian age history was regularly exploited as a polemical tool designed to give a set of interpretations or values, a process evident in this heroism script.^143^ The aspiration implicit in foregrounding ‘sincere devotion to the cause’ aired a role model of values for readers supportive of mental and moral recovery. A lecture titled *On the Elements of Success in Life* was presented at the asylum and reported in the *Mirror*, again connecting with a moral vein celebrated through the asylum’s Library Club, which met amongst shelves of books and periodicals.^144^ Another piece was titled *Reflections on Society, Ancient and Modern*.^145^ Moralising through writing similarly pervaded *The Edinburgh Review* (1802–1929), a periodical described as ‘one of those social vehicles of improvement and enlightenment that flourished throughout Europe’.^146^ Volumes held in the asylum reading room included Captain James Cook’s voyages of discovery journals (1768–77) and Gibbon’s *Decline and Fall of the Roman Empire (*1776–88). As a historian of faith, Gibbon’s Decline and Fall is dense with moral and civilizational themes suited to moral treatment and designed to entertain educated readers.^147^ A contributor to the *Mirror’s* October 1851 edition, in a piece titled ‘Well Meaning Men’, conveys an assessment of ‘a species of character’ whose activities do more harm than good, ‘a species of character that delights in going about and doing things that, though done with the best intentions, do not invariably turn out to be good, but very frequently the counterpart thereof … The world is in fact overrun with well-meaning men’.^148^

Judgemental prose essays abound in the *Mirror*, often taking the form of extensive commentaries about military conflict and shifting national alliances. On other pages, local narratives present stories of wrongdoing, the clandestine behaviour projecting a moral warning to readers and an opportunity to highlight wider duplicity. Writers for the *Mirror* would acquire news from the many newspapers in the Asylum Library. One favourite theme involved concealed identities. The editor of the *Mirror* may have encouraged patients to turn their gaze from the asylum interior to wider society to stitch them into the mainstream. A failure of character is illustrated through the next *Mirror* extract with a theme of deception and abuse of trust. The incident is the swindling a city jeweller of a diamond ring in the city of Liverpool:

*               SWINDLING*

Mr__ is a gentleman well known in Europe and America for his comprehensive philanthropy, and in one of his numerous journeys he encountered a gentleman... The two entered into amicable conversation, and everything connected with pursuits of society, past, present, and future were discussed by them. This gentleman informed Mr__ that in England an extremely organised system of swindling existed which was too often countenanced by men in the highest circles of socie

The piece proceeds to describe how a traveller of most respectable appearance swindled a Liverpool jeweller by tricking him into purchasing a diamond ring that he discovered the next day to be a ‘worthless bauble’. The jeweller pursued the thief to his hotel but found he had left by one of the ‘earliest stages for London’.^149^ Another contemporary case, of the Victorian gentleman William Wilshin, also represents a duplex identity of the respectable gentleman and habitual criminal which this patient understands as being widespread. Crime historians have used his case to examine ideas of class and respectability in Victorian England, a nexus exploited by the fraudster and, they suggest, Britain’s industrial entrepreneurs involved in commerce.^150^ The middle-class gentlemanly appearance could conceal truth beneath norms of respectability; character could be fabricated. R. L. Stevenson’s novel *The Strange Case of Dr. Jekyll and Mr. Hyde* (1886) embroiders personality disorder with deception and violence in parallel with a normal persona. Victorian thinkers postulated that the ‘split’ personality was a common condition.^151^

While the design of the Edinburgh asylum environment created a dramaturgical encryption of gentlemanly class normality, the realities of Victorian psychiatric care in the lunatic asylum also contained a harshly repressive environment. The Scottish Lunacy Commission 1857 observed that in lunatic asylums the straight-waistcoat, or the straps, or muffs were almost entirely banished from Chartered asylums, ‘but we have reason to think that seclusion for long periods is frequently used, some for several months in cell-like accommodation with stone floors and darkened windows. Loose straw scattered on the floor was the patient’s bedding’.^152^

Victorian Gothic literature treated asylums as ghostly habitations where truth could be locked away.^153^ The Physician’s Annual Report of 1860^154^ described the arrival at the asylum of some patients bearing the warrants for their own admission, and in one case the patient ‘had been led to believe he was bearing a warrant for his wife’s detention, to whom he introduced me, with a wink and a nod of caution…It was with some difficulty I could persuade him I had not made a mistake in detaining him, and certainly the deception by which he was tricked into the Asylum was by no means conducive to his subsequent contentment or cure’. Commenting publicly upon problems about gaining accurate information about a patient’s psychological history, the Physician noted careful attempts by 62 families, ‘to conceal the existence of the malady in other members or ancestors of the family, and in particular to ignore peculiarities and eccentricities, amounting in many instances to proofs of insanity’.^155^

## Conclusion

*The Morningside Mirror* can be understood as a rhetorical device designed to undermine the stigma of ‘madness’ and social meanings at large about lunatic asylums. During the period of its publication only a relatively small number of individuals would have known about other dimensions of asylum life that have been described in this article through references to primary sources. The various contributions to debate and worlds of feeling in the periodical are likely to have been assimilated by readers as representative of the asylum’s interior and the efficacy of moral treatment. From the perspective of psychological medicine, the *Mirror* was a method of scientific communication in an informal literary mould. Writers for the *Mirror* claimed a space in society which they would otherwise not have experienced in the incarcerating regime of the asylum. The forms of knowledge the *Mirror* documents stand in contrast to the diagnostic briefs of reductive psychopathological labels. Humanising, as opposed to de-humanising, is a project that can be attributed to the Asylum’s political and therapeutic initiatives behind the *Mirror’s* rationale.

The Scottish *literati* believed that art had a moral function, that its aesthetic terminology constituted a moral discourse. Cultivation, taste, tradition and experience evoked values of beauty, goodness and truth.^156^ One correspondent, whose view was published in the *Mirror*, defended the authenticity of the periodical against aristocratic derision that had been published in the *Edinburgh Evening News*.^157^ A newspaper in Hereford, England, drew attention to pieces by a patient in the Edinburgh Royal Asylum whose work challenged the stigma of insanity and the poem *Sunset* by a ‘Lunatic Poet’, first published in the *Mirror*, was reprinted in the *Hereford Journal*. An English journalist remarked that among the four hundred persons confined there are those with literary talent who might ‘take their station with those deemed of perfect and sound mind’.^158^ Besides its moral function, the *Mirror* endorsed feeling, belonging and human connection amidst a climate of separation and loss.

Thomas Mayo (1790–1871), author of medical texts and consultant-physician at Ticehurst personified the humanistic underpinning of a moral therapist.^159^ Mayo held an Oxford University degree in *Literae Humaniores* and was on friendly terms with eminent people dedicated to education and reform, including John Keble (1792–1866) and Thomas Arnold (1795–1842). Mayo appears as a trailblazer for *The Morningside Mirror’s* incorporation of a self-consciously moral culture.^160^ Charlotte Mackenzie’s study of Ticehurst private asylum for upper-class patients highlights cultivation opportunities. Patients are imagined as alert to re-experiencing the traditions of classist erudition.^161^

Albert Pionke describes the works by authors Thackery, Trollope, Dickens and George Eliot in relation to social rituals that flourished amongst professionalism’s culture of literary representations.^162^  *The Morningside Mirror*, whilst appearing to embody a conservative literary culture, presented a varied range of content, some critical in its purchase on the harms of incarceration and forms of social injustice. Conditions in the asylum may have aggravated melancholia: W.A.F. Browne, Lunacy Commissioner, reported in 1860 that the Register of Seclusions ‘contains a very large number of entries’.^163^ Nevertheless, mental health therapists have since adopted expressive writing as a treatment; putting upsetting experiences into words is now a recognised therapeutic intervention to relieve mood disorders. Scientific studies demonstrate this activity supports executive functioning and relieves depressive mood.^164^ Alan Beveridge, a practicing psychiatrist commenting on the Edinburgh Asylum’s patients, argues that letter writing about their mental breakdown was ‘a lifeline to sanity’; the act of writing assisted them to come to terms with abnormal experiences conjuring ‘strange worlds’.^165^ The asylum’s Musical Association, ran lecture series such as ‘On Discovery in Africa’, exploring Stanley and Livingston’s exploits and moral characters and ‘Aspects of Self-Sacrifice’ which proffered ‘many wise observations and pious reflections’.^166^ The Victorian virtues of ‘Civilisation’ exist in value judgements conveyed by resident-physicians about the demography of the insane occurring less frequently ‘where the mental powers have been more called into action’.^167^ Resident asylum superintendent Dr.Skae’s theory regarding insanity’s national increase blamed it on the rapid pace in industrialisation ‘deranging the healthy equilibrium which binds the faculties together’.^168^

The *Mirror* participates in quietly asking us to think about human normality. It is hoped that this article contributes to filling a lacuna in our knowledge of this Scottish asylum. Moral treatment’s spirit of optimism existed prior to biological psychiatry’s scientific methods of enquiry. That methodology established a globally reputable evidence base about the deterministic roles of biological and genetic factors in mental disease. Replicable scientific methods nurtured the formulation of organic disease models of insanity, initiating a shift away from a more serendipitous and charisma-led intervention associated with the personalities of resident-physicians to an impersonal therapeutic model recognising and debating biological phenotypes in the pages of specialist international journals.^169^ To a perhaps surprising extent, however, some therapeutic strategies have barely changed since the first issue of *The Morningside Mirror*.

## Footnotes

1 A. Mizel, ‘Righteous Restraint in *Hard Times* and *Jane Eyre*’, *Renaissance: Essays on Values in Literature*, 2016, 68, 176–92.

2 M. Faubert, ‘Cure, Classification, and John Clare’, *Victorian Literature and Culture*, 2005, 33, 269–91, 273.

3 L. McDaniel, ‘The Manic-Depressive Father in George Meredith’s *Harry Richmond*’, *Victorian Literature and Culture*, 2015, 43, 589–605, 603.

4 S. A. Hill and R. Laugharne, ‘Mania, Dementia and Melancholia in the 1870s: Admissions to a Cornwall Asylum’, *Journal of the Royal Society of Medicine*, 2003, 96, 361–63, 361.

5 K. S. Kendler, ‘The Genealogy of the Clinical Syndrome of Mania: Signs and Symptoms Described in Psychiatric Texts from 1880 to 1900’, *Psychological Medicine*, 2018, 48, 1573–91.

6 D. Bhugra, ‘Alienated Alienists: A New Hope?’, *The Lancet Psychiatry*, 2014, 1, 257–59.

7 M. Gelder, P. Harrison and P. Cowen, eds*, Shorter Oxford Textbook of Psychiatry*, 5th edn (Oxford: Oxford University Press, 2006), 4.

8
*The Morningside Mirror* Lothian Health Services Archive, University of Edinburgh (henceforth MM), 15 December 1845. The first edition of the *The Morningside Mirror* periodical was published in 1845 in the Asylum and it continued as a monthly periodical; collected editions in bound volumes are available in the University of Edinburgh special collections library archive located within the Main Library in George Square, Edinburgh, under reference GB239 LHB7 Royal Edinburgh Hospital: History and Publications.

9 B. Edginton, ‘A Space for Moral Management: The York Retreat’s influence on Asylum Design,’ in L. Topp, J. E. Moran and J. Andrews, eds, *Madness, Architecture and the Built Environment: Psychiatric Spaces in Historical Context* (London: Routledge, 2007), 85–104.

10 MM, 15 October 1846.

11 M. Barfoot, ‘David Skae: Resident Asylum Physician; Scientific General Practitioner of Insanity’, *Medical History*, 2009, 53, 469–88.

12 MM, 5 January 1853, 42.

13 The ‘western department’ housed poor and pauper patients, and an ‘immediate class of private patients’ who, like wealthy private patients, had access to outings which they could then describe in *The Morningside Mirror*. Annual Report, 1880, 22. https://wellcomecollection.org/works/.

14 MM, 15 February 1871, 33: Account of a lecture presented in the Asylum on Sir Isaac Newton. And see MM, 15 August 1860, 11, and MM, 15 June 1861, 68.

15 Diarmid A. Finnegan, ‘“An Aid to Mental Health”: Natural History, Alienists and Therapeutics in Victorian Scotland’, *Studies in History and Philosophy of Science. Part C, Studies in History and Philosophy of Biological and Biomedical Sciences*, 2008, 39, 326–37.

16 Report respecting the lunatic asylum of Edinburgh for 1852, 25. WLM.28.BS2E23. Wellcome Collection. https://wellcomecollection.org/works/. Annual Reports of the Edinburgh Asylum henceforth referenced as ‘AR’.

17 J. Laws, ‘Crackpots and Basket-Cases: A History of Therapeutic Work and Occupation’, *History of the Human Sciences*, 2011, 24, 65–81, 67.

18 K. Doerner, *Madmen and the Bourgeoisie: A Social History of Insanity and Psychiatry* (Oxford: Basil Blackwell, 1981), 59.

19 R. R. Grinker, ‘Autism, “Stigma,” Disability: A Shifting Historical Terrain’, *Current Anthropology*, 2020, 61, 55–67.

20 Barfoot, ‘David Skae’, 485.

21 Emma Middleton, ‘From Manacles to Management: Moral treatment and the Morningside Mirror 1845–1855’, University of Edinburgh Conference Paper, *Society for the Social History of Medicine*; V. Long, *Destigmatising Mental Illness: Professional Politics and Public Education in Britain 1870–1970* (Manchester, Manchester University Press, 2014).

22 Kelly J. Mays, 'The Publishing World.' P. Brantlinger and W. Thesing, eds, *A Companion to the Victorian Novel* (Malden, MA: Blackwell, 2005), 21–23.

23 J. R. Edwards and S. P. Walker, ‘Lifestyle, Status and Occupational Differentiation in Victorian Accountancy’, *Accounting, Organizations and Society*, 2010, 35, 2–22.

24 C. A. Beardmore, ‘Death, Grief and the Victorian GP: A Case Study of Edward Wrench of Baslow, Derbyshire, 1862–1898’, *Midland History*, 2022, 47, 313–30, 329.

25 L. P. Karpenko and S. R. Claggett, eds, *Strange Science: Investigating the Limits of Knowledge in the Victorian Age* (Ann Arbor: University of Michigan Press, 2017).

26 J. Misbach and H. J. Stam, ‘Medicalizing Melancholia: Exploring Profiles of Psychiatric Professionalization’, *Journal of the History of the Behavioral Sciences*, 2006, 42, 41–59, 50.

27 A. Black, ‘Lost Worlds of Culture: Victorian Libraries, Library History and Prospects for a History of Information’, *Journal of Victorian Culture,* 1997, 2, 95–112, 99.

28 H. O’Neill, ‘John Stuart Mill and the London Library: A Victorian Book Legacy Revealed’, *Book History*, 2016, 19, 256–83, 256.

29 D. Hooper, ‘The St. Andrew’s Graduates’, *British Medical Journal*, 1867, 2, 118.

30 Gillian Williamson, *British Masculinity in the ‘Gentleman’s Magazine’ 1731–1815* (UK: Palgrave MacMillan, 2015), 3.

31 M. De Waard, ‘History’s (Un)reason: Victorian Intellectualism from J.S. Mill to Leslie Stephens’, *Victorian Studies*, 2011, 53, 457–67.

32 B. Jenkins, ‘David Brewster at the Royal Society of Edinburgh: Politics and Patronage in Scotland, 1808–37’, *The Scottish Historical Review*, 2022, 255, 20–45.

33 J. R. Davis and A. Nicolls, ‘Fredrich Max Muller: The Career and Intellectual Trajectory of a German Philologist in Victorian Britain’, *Publications of the English Goethe Society*, 2016, 85, 67–97.

34 I. Crawford, ‘Faithful Sympathy: Dickens, the Edinburgh Review, and Editing Household Words’, *Victorian Periodicals Review*, 2011, 44, 42–68. P. E. Dumas, ‘The Edinburgh Review, the Quarterly Review, and the Contributions of the Periodical to the Slavery Debates’, *Slavery and Abolition*, 2017, 38, 559–76.

35 A. Bautz, *The Reception of Jane Austen and Walter Scott: A Comparative Longitudinal Study* (London: Bloomsbury, 2007), 82.

36 Megan Coyer, *Literature and Medicine in the Nineteenth-Century Periodical Press: Blackwood’s Edinburgh Magazine, 1817–1858* (Edinburgh: Edinburgh University Press, 2017).

37 Patrick M. Brantlinger, ed, *Energy and Entropy: Science and Culture in Victorian Britain* (Bloomington, IN: Indiana University Press, 1989).

38 MM, 15 January 1848, 14.

39 MM, 15 February 1883, 13–14.

40 Natalie Mera Ford, ‘Placing Literature in Nineteenth-Century British Psychology’, Critical Survey, 27, 2015, 4–20.

41 AR, 1840, 13.

42 A. Clark, ‘The Rhetoric of Chartist Domesticity: Gender, Language, and Class in the 1830s and 1840s’, *The Journal of British Studies*, 1992, 31, 62–88, 66.

43 Seth Koven, 'The "New Man" in the Slums: Religion, Masculinity, and the Men's Settlement House Movement,'.*Slumming: Sexual and Social Politics in Victorian London*. STU-Student edn (Princeton NJ: Princeton University Press, 2006), 228–281.

44 M. A. Kimball, ‘London through Rose-Colored Graphics: Visual Rhetoric and Information Graphic Design in Charles Booth’s Maps of London Poverty,’ *Journal of Technical Writing and Communication*, 2006, 36, 353–81.

45 Scott Orford *et al.* ‘Life and Death of the People of London: A Historical GIS of Charles Booth’s Inquiry,’ *Health Place*, 2002, 8, 25–35; Paul Kahn, ‘Data storytelling’ in late Victorian London: Review of Mary S. Morgan et al, *Charles Booth’s London Poverty* Maps (London: Thames and Hudson: 2019). *Eye Magazine*, 2021, 101(26).

46 A. Wilkinson, ‘A Middle-Class Community? Social Structure in Victorian Roundhay, Leeds, 1851–1891’, *Family and Community History*, 2002, 5, 5–18, 132.

47 AR, 1888, 9–10.

48 AR, 1888, 14.

49 A. Vrettos, 'Victorian Psychology,' Patrick Brantlinger and William B. Thesing, *A Companion to the Victorian Novel* (London: Blackwell, 2005), 67–83.

50 J. Forbes, *Of Nature and Art in the Cure of Disease* (London: John Churchill, 1857).

51 G. Davis, *‘The Cruel Madness of Love’: Sex, Syphilis and Psychiatry in Scotland, 1880–1930* (Amsterdam: Brill Academic Publishers, 2008), 44, 227.

52 A. Beveridge and D. Cunynghame, D. (2021) ‘“A Bruised Reed Shall He Not Break”: John Miles’s Portraits of Patients at the Royal Edinburgh Asylum. Part 1’, *The Journal of the Royal College of Physicians of Edinburgh*, 2021, 51, 298–308, 414.

53 A. Owen, R. Fyson, and S. Roberts, eds., *The Duty of Discontent: Essays for Dorothy Thompson* (London: Mansell, 1995), 242–43.

54 AR, 1836, 8.

55 AR, 1849, 16.

56 AR, 1853, 1867, 1876, 1880.

57 AR, 1880, 9.

58 Royal Edinburgh Hospital General Register, 25 Sep. 1851–31 Dec. 1857, LHB7/35/1 and March 1863, LHB7/35/2.

59 AR, 1880, 40.

60 AR, 1841, 15.

61 AR, 1841, 17.

62 AR, 1849, 41.

63 AR, 1849, 17.

64 AR, 1849, 69.

65 R. Golding, 2021, 46.

66 W. A. F. Browne ‘The Moral Treatment of the Insane: A Lecture,’ *The British Journal of Psychiatry*, 1864, 10, 309–37.

67 Campbell, 2017, 34.

68 AR, 1848, 25.

69 AR, 1880, 62.

70 J. Andrews, ‘Death and the Dead-House in Victorian Asylums: Necroscopy Versus Mourning at the Royal Edinburgh Asylum, c. 1832–1901’, *History of Psychiatry*, 2012, 23, 6–26, 17.

71 Robert Douglas-Fairhurst, *Victorian Afterlives: The Shaping of Influence in Nineteenth-Century Literature* (Oxford: Oxford University Press, 2002).

72 Campbell 2017, 48; A. Scull, *Hysteria: The Biography* (Oxford: Oxford University Press, 2009).

73 AR, 1850, 10.

74 AR, 1850, 11.

75 Barfoot, ‘David Skae’, 486.

76 LHB7/35/1 and 2 Edinburgh Royal Asylum Register 1817–1863.

77 MM, 1845, 2.

78 MM, 15 June 1846, 80.

79 AR 1853, 32.

80 AR 1853, 13.

81 MM, 15 October 1851, Editorial.

82
*Ibid*.

83 S. D. Walbaum, ‘The Invisible Woman: Susan Carnegie and Montrose Lunatic Asylum’, *History of Psychiatry*, 2019, 30, 409–23.

84 A. Aspinall, ‘The Social Status of Journalists at the Beginning of the Nineteenth Century’, *The Review of English Studies*, 1945, XXI(83), 216–32, 220–21.

85 AR, 1852, 29, 36.

86 AR, 1849, 30.

87 AR 1880, 22.

88 AR 1880, 51.

89 AR, 1851, 32.

90 AR, 1850, 35.

91 Report by Her Majesty’s Commissioners appointed to inquire into the state of lunatic asylums in Scotland: And the existing law in reference to lunatics and lunatic asylums in that part of the United Kingdom (Edinburgh: HMSO, 1857), 95, 90. Wellcome Collection M21286.

92 C. Ledrum ‘“Periodical Performance”: The Editor Figure in Early Nineteenth Century Literary Magazines’ (PhD. Thesis, University of Ottawa: ProQuest Dissertations Publishing, 2010) NR73922.

93 MM, 15 October 1846, 6.

94 L. O’Hagan, ‘The Dip Pen as a Source of Social Distinction in Victorian Britain’, *History of Retailing and Consumption*, 2018, 4, 187–216.

95 AR, 1841, 8.

96 MM, August 1846, Preface.

97 Tait’s Edinburgh Magazine 12: 143. Published William Tait, 107, Prince’s Street, Edinburgh. Downloaded at: ProQuest British Periodicals Collection 2 17/12/16.

98 P. France, ‘Looking Abroad: Two Edinburgh Journals in the Early Nineteenth Century’, *Forum for Modern Language Studies*, 2010, 46, 2–14, 4.

99 T. Joseph, ‘A Choice of Pseudonyms,’ *Library Review*, 1974, 24, 204–6.

100 Hannah Barker, *Newspapers, Politics and English Society 1695–1855* (London: Routledge, 2000) 25–26.

101 Gayle Davis, ‘Sir Thomas Clouston (1840–1915)’, *Nature*, 1940, 145, 619. https://doi.org/10.1038/145619c0

102 AR, 1840, 14.

103 Long, *Destigmatising Mental Illness*, 30.

104 A. Janson, ‘From Statistics to Diagnostics: Medical Certificates, Melancholia, and “Suicidal Propensities” in Victorian Psychiatry’, *Journal of Social History*, 2013, 46, 716–31.

105 AR, 1853, 23.

106 AR, 1853, 12.

107 AR, 1852, 1867, 1876, 1880.

108 AR, 1853, 10.

109 E. Monzingo and D. Shanahan, ‘The Expression of Self and Grief in the Nineteenth Century: An Analysis through Distant Readings’, *Nineteenth-Century Music Review*, 2021, 18, 83–107, 85.

110 Monzingo and Shanahan, ‘The Expression of Self’, 83.

111
*Ibid.,* 85.

112 Janson, ‘From Statistics to Diagnostics’, 72.

113 AR, 1849, 21.

114 MM, 15 July 1847, 80.

115 MM, 15 July 1846, 57–58.

116 MM, 22 October 1849, 119; MM, 15 Nov 1849, 131; MM, 15 January 1858, 26.

117 M. Gold, ‘“Frames of reference” in Victorian England: What Elizabeth Gaskell’s *North and South* Reveals about Perceptions of the Employment Relationship’, *Journal of Industrial Relations*, 2021, *63*, 126–48, 127.

118 D. R. Green, ‘Pauper Protests: Power and Resistance in Early Nineteenth-Century London Workhouses’, *Social History*, 2006, 31, 137–59.

119 Daniel Layman, *Locke Among the Radicals: Liberty and Property in the Nineteenth Century*, online edn (New York: Oxford Academic, 2020).

120 J. R. Edwards, ‘Accounting, Publicity and Class Conflict in Victorian Britain’, *Accounting and Business Research*, 2022, 52, 321–46, 327.

121 John H. Hagan, ‘The Poor Labyrinth: The Theme of Social Injustice in Dickens’s “Great Expectations”,’ *Nineteenth-Century Fiction*, 1954, 9, 169–78, 173.

122 B. Disraeli, *Sybil: or The Two Nations (London: Henry Colburn, 1845).*

123 Elizabeth Gaskell, *North and South* (London: Penguin Books, 2003 [1855]).

124 Richard Gravil, *Reading ‘Bleak House’.* (Penrith, CA: HEB Humanities E-Books, 2012) 32–33; J. Foster, ‘Bureaucratic Sensibility: Bleak House as a Layperson’s Guidebook to Officialdom’, *Dickens Quarterly*, 2022, 39, 24–41.

125 G. Vargo, ‘“Outworks of the Citadel of Corruption”: The Chartist Press Reports the Empire’, *Victorian Studies*, 2012, 54, 227–53.

126 P. J. Cain, ‘Empire and the Languages of Character and Virtue in Later Victorian and Edwardian Britain,’ *Modern Intellectual History*, 2007, 4, 249–73.

127 Carl J. Griffin, *The Politics of Hunger: Protest, Poverty and Policy in England, c. 1750–c. 1840*. (Baltimore, Maryland: Project Muse, 2020).

128 MM, 15 Sept 1847, 93.

129 MM, 15 April 1847, 53.

130 AR, 1880, 4.

131 A. McNee, ‘Mountaineering Accidents’, *Victorian Review*, 2014, 40, 26–30, 26.

132 Beveridge, *Life in the Asylum. See also* J. Girardin, *Travel in The Alps: The Construction of a Transnational Space Through Digital and Mental Mapping, 1750–1830* (unpublished PhD thesis, University of St. Andrews, 2016). http://research-repository.st-andrews.ac.uk/.

133 Susan M. Ryan, 'Introduction: Moral Markets,' *The Moral Economies of American Authorship: Reputation, Scandal, and the Nineteenth-Century Literary Marketplace* (Oxford: Oxford University Press, 2016), 6.

134 Royal Edinburgh Hospital Case Book, *5*, January 1846–December 1854, 312–15.

135 Royal Edinburgh Hospital Case Book, (5), January 1846–December 1854, 312–15.

136
*Ibid*., 312.

137
*Ibid*., 313.

138 MM, 16 Oct. 1848, 1, 1–6.

139 R. Bellon, ‘“There is Grandeur in this View of Newton”: Charles Darwin, Isaac Newton and Victorian Conceptions of Scientific Virtue’, *Endeavour*, 2014, 38, 222–34.

140 Matthew Sussman, ‘Stylistic Virtue in Nineteenth-Century Criticism’, *Victorian Studies*, 2014, 56, 225–49.

141 MM, 15 November 1845, 23–24.

142 J. Coleman, ‘The Scottish Covenanters’, in G. Atkins, ed, *Making and Remaking Saints in Nineteenth Century Britain* (Manchester: Manchester University Press, 2016) 177–93.

143 Michael J. Turner, ‘Defending “the Principle of Representation”: Andrew Bisset, The English Civil War, and *The History of the Struggle for Parliamentary Government in England’*, *Journal of Victorian Culture*, 2015, 20, 531–48.

144 MM, 15 February 1878, 12.

145 MM, 20 November 1865, 40.

146 J. Lomonaco, ‘Adam Smith’s “Letter to the Authors of the Edinburgh Review”’, *Journal of the History of Ideas*, 2002, 63, 659–76, 659.

147 G. Quilley, ‘Re-enacting Cook’s Voyages in Nineteenth-Century Visual Culture’, *Studies in English Literature*, 2016, 56, 845–69; P. Cosgrove, ‘The Circulation of Genres in Gibbon’s Decline and Fall of the Roman Empire’, *ELH John Hopkins University Press*, 1996, 63, 109–38; D. Wootton, ‘Narrative, Irony, and Faith in Gibbon’s Decline and Fall’, *History and Theory: Studies in the Philosophy of History*, 1994, 33, 77–105.

148 MM, VII(1) 1851, 1. The contributing author signed as ‘HOM’.

149 MM, 15 Dec 1845, 38–40.

150 G. N. Woolnough, ‘A Victorian Fraudster and Bigamist: Gentleman or Criminal?’ *Criminology and Criminal Justice*, 2019, 19, 439–55.

151 B. Wall, ‘Inheritance and Insanity: Transatlantic Depictions of Property and Criminal Law in Nineteenth Century Scottish and American Fiction’ (unpublished Ph.D. thesis, University of Edinburgh, 2015), 166.

152 Royal Lunacy Commission, 1857, 84–85.

153 B. E. Noad, ‘Gothic Truths in the Asylum’, *Gothic Studies*, 2019, 21, 176–90.

154 AR, 1860, 22.

155 AR, 1860, 23.

156 Margaret Watkins, ‘Review of Leslie Ellen Brown’s Artful Virtue: The Interplay of the Beautiful and the Good in the Scottish Enlightenment,’ *Scottish Historical Review*, 2020, 99, 154–55.

157 ‘A Lunatic’s Reply to Lord Rosebery’ *Edinburgh Evening News* (Edinburgh, 15 Dec 1906), 6. *British Library Newspapers* accessed 22 Dec. 2019, Gale Document No. JE3240029469.

158 ‘Arts and Popular Culture,’ *Hereford Journal* (Hereford, England 21 Jan. 1846), 4. *British Library Newspapers* accessed 22 Dec. 2019. Gale Document No. GW3222190632.

159 Charlotte Mackenzie, 'The Asylum and Moral Reform,' *Psychiatry for the Rich: A History of Ticehurst Private Asylum 1792–1917* (London: Routledge, 1992), 70–105.

160 Charlotte Mackenzie, *A Family Asylum: A History of the Private Madhouse at Ticehurst in Sussex, 1792–1917* (Doctoral thesis 1986, UCL, London), 2.

161 Mackenzie, ‘*A Family Asylum*’.

162 A. D. Pionke, *The Ritual Culture of Victorian Professionals: Competing for Ceremonial Status, 1838–1877*. 1st edn (Farnham: Routledge, 2013).

163 AR, 1860, 9.

164 M. Reinhold, P. C. Bürkner, and H. Holling, ‘Effects of Expressive Writing on Depressive Symptoms—A Meta‐Analysis’ *Clinical Psychology: Science and Practice*, 2018, 25, e12224; J. Qian, X. Zhou, X. Sun, M. Wu, S. Sun, and X. Yu, ‘Effects of Expressive Writing Intervention for Women’s PTSD, Depression, Anxiety and Stress Related to Pregnancy: A Meta-Analysis of Randomized Controlled Trials’, *Psychiatry Research*, 2020, 288, 112933.

165 A. Beveridge, ‘Voices of the Mad: Patients’ Letters from the Royal Edinburgh Asylum, 1918–1908’, *Psychological Medicine*, 1997, 27, 899–908, 901.

166 MM, 15 March 1876, 19.

167 Royal Lunacy Commission, 1857, 39.

168 AR, 1852, 25.

169 V. Hess and J. A. Mendelsohn, ‘“Sauvages” Paperwork: How Disease Classification Arose from Scholarly Notetaking’, *Early Science and Medicine*, 2014, 19, 471–503.

